# Adenovirus-mediated co-expression of the TRAIL and HN genes inhibits growth and induces apoptosis in Marek’s disease tumor cell line MSB-1

**DOI:** 10.1186/s12935-015-0172-6

**Published:** 2015-02-18

**Authors:** Dongxiao Dong, Jing Gao, Ying Sun, Yuqing Long, Meng Li, Dongchao Zhang, Jianfang Gong, Liang Xu, Liuan Li, Shunyi Qin, Jifei Ma, Tianming Jin

**Affiliations:** College of Animal Sciences and Veterinary Medicine, Tianjing Agricultural University, Tianjing, 300384 China; Tianjing Shenji Group Co., Ltd, Tianjing, 300380 China; Tianjing Ruipu Group Co., Ltd, Tianjing, 300380 China

**Keywords:** Recombinant adenovirus, Tumor necrosis factor (TNF) related apoptosis inducing ligand, Hemagglutinin-neuraminidase, MSB-1 cell line, In vitro tumor-inhibiting effect

## Abstract

**Background:**

The objective of this study was to determine the in vitro tumor-inhibitory effect of a recombinant adenovirus expressing a fusion protein of tumor necrosis factor (TNF) related apoptosis inducing ligand (TRAIL) and hemagglutinin-neuraminidase (HN) genes on the MSB-1 Marek’s disease tumor cell line.

**Methods:**

TRAIL and HN genes were amplified from lymphocytes in the peripheral blood of chickens and the LaSota strain of Newcastle disease virus (NDV), respectively, using RT-PCR. The two genes were connected with a 2A connecting peptide by site-directed mutagenesis and gene splicing by overlap extension (SOE). The target gene TRAIL-2A-HN was cloned into the shuttle vector pShuttle-CMV. Homologous recombination was carried out with the vector pAdeasy-1 in the bacterium BJ5183 to construct the recombinant adenovirus plasmid pAd-TRAIL-2A-HN. After linearization, the plasmid was transfected into AD293 cells and packaged. Real-time quantitative PCR (RT-PCR) and fluorescence microscopy confirmed the introduction of the recombinant adenovirus into AD293 cells. The TCID_50_ method (50% tissue culture infectious dose) was employed to determine viral titers for the exprimental and control viruses, which met criteria for use. The Marek’s disease tumor cell line MSB-1 was transfected with the constructed recombinant adenovirus. The infectivity of the recombinant adenovirus and the expression levels of exogenous genes were detected with RT-PCR and western blotting. The effects of the recombinant adenovirus on the growth of MSB-1 cells and cellular apoptosis were determined using flow cytometry.

**Results:**

The recombinant adenovirus infected the cultured cells in vitro, and replicated and expressed exogenous genes in the cells. The recombinant adenovirus Ad-TRAIL-2A-HN inhibited the growth of MSB-1 cells and induced apoptosis by expressing exogenous genes. The rate of induced MSB-1 cell apoptosis reached 11.61%, which indicated that TRAIL and HN produced synergistic tumor-inhibiting effects.

**Conclusion:**

The constructed TRAIL-2A-HN fusion gene combined the apoptosis-inducing function of TRAIL and the adsorptive capacity of HN from NDV for tumor cells, and the capacity of the recombinant adenovirus expressing this fusion gene to induce tumor cell apoptosis was reported. These results provide a basis for future in vivo tumor suppression studies using recombinant adenoviruses.

## Background

Marek’s disease (MD) is a lymphoproliferative infectious disease in chickens that is caused by the Marek’s disease virus (MDV). Since the first report of MD by Joseph Marek in 1907, the pathogenicity of MDV has progressed from moderate virulence (mMDV), to strong virulence (vMDV), to very strong virulence (vvMDV). At the beginning of the 1980s, a mutant with super strong virulence (vv + MDV) appeared [1]. This disease is common in poultry farms with intensive and condensed rearing, and it is one of the major neoplastic diseases endangering poultry breeding. Clinically, MD is generally prevented and controlled by vaccination. However, significant technical issues remain with regard to the prevention and control of neoplastic diseases. Emerging gene testing and therapy methods have provided new strategies for the treatment of neoplastic diseases that solve some of these issues.

In gene therapy, external normal genes are introduced into the target cells to rectify or compensate for the effects of genetic defects and abnormalities and achieve therapeutic goals. Currently, adenovirus (Ad) is used as the vector through which external genes are introduced in 40% of clinical tests related to gene therapy. Gene therapy using adenovirus vectors is one of the most promising gene transfer methods in gene therapy, and this method has shown non-toxicity and non-diffusibility in animal tests [2]. The successful construction of human adenovirus (i.e. Ad5) vectors and their clinical application has resulted in the adenovirus becoming the most common means through which therapeutic genes are introduced into target cells [3,4].

Tumor necrosis factor (TNF) related apoptosis inducing ligand (TRAIL), or Apo2 ligand (Apo2L), is a member of the TNF super-family cloned from the cDNA library of human myocardium by Wiley et al. in 1995 [5], which shows high homology with Fas ligand (FasL). It is so named because its amino acid sequence has the predicted structural characteristics of the TNF super-family and it can induce apoptosis in human lymphocytes transformed by Jurkat cells and EB viruses.

TRAIL can selectively kill tumor cells in a manner that is minimally virulent to most normal cells. Therefore, it is a focus of tumor treatment research. Some researchers have achieved tumor cell apoptosis by promoting or enhancing TRAIL expression in tumor cells. In these studies, adenovirus recombinant vectors carrying a gene for functional TRAIL were constructed and introduced into tumors such as human renal carcinoma [6], breast carcinoma [7], hepatic carcinoma [8], and squamous carcinoma [9], in which the functional TRAIL protein was continuously expressed to inhibit tumor growth.

Cassel et al. showed in 1965 that the outbreak of Newcastle disease (ND) inhibited metastasis in patients with advanced gastric carcinoma [10]. After this report, more attention was focused on the anti-tumor effect of Newcastle disease virus (NDV), and research of this type is currently in the clinical stage. Hemagglutinin-neuraminidase (HN) is the primary component of the large spike glycoprotein on the NDV envelopes, has a length of 1734 bp (encoding 577 amino acids) and a molecular weight of 63 kD, and can control hemagglutinin and neuraminidase activity. Hemagglutinin is responsible for recognizing the sialic acid-containing receptors of permissive cells. Neuraminidase can hydrolyze sialic acid on the surface of host cells, decompose sialic acid-containing receptors, expose the biological recognition sites of host cells, promote the release of new virus particles from the infected cell membrane, and finally induce TRAIL expression by mononuclear lymphocytes in the peripheral blood [2,11,12]. To enhance the inhibitory effect of TRAIL on MD tumor cells, it was introduced in combination with HN in this experiment.

The TRAIL and HN genes were amplified from the lymphocytes in the peripheral blood of chickens and the NDV (LaSota lentogenic strain), respectively. A recombinant adenovirus (Ad-TRAIL-2A-HN) with a fusion protein of the above genes was constructed to take advantage of the targetability of TRAIL to tumor cells, the apoptosis-inducing effect of HN, and the efficient and stable expression of exogenous genes by adenovirus. The in vitro inhibitory effect of Ad-TRAIL-2A-HN on Marek’s disease tumor cell line MSB-1 was evaluated to explore the ability of recombinant adenoviruses to induce apoptosis of MSB-1 tumor cells and thus their potential use in clinical gene therapy.

## Results

### PCR identification of TRAIL, HN, and TRAIL-2A-HN

**Figure 1:**
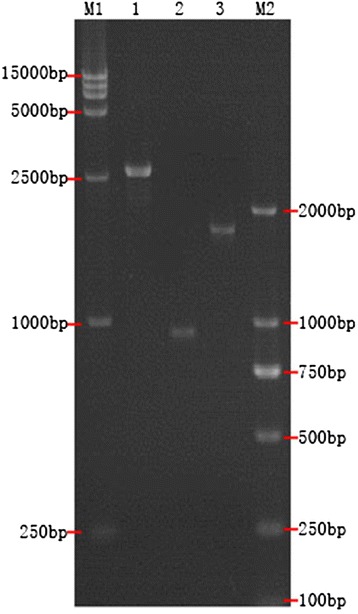
**PCR Amplification results for TRAIL, HN, and the TRAIL-2A-HN fusion gene.** M1: DL15000 DNA Marker; 1: SOE product of TRAIL-2A-HN fusion gene; 2: PCR product of TRAIL gene; 3: PCR product of HN gene; M2: DL2000 DNA Marker.

Using PCR, 915 bp of the TRAIL gene and 1716 bp of the HN gene were amplified. The TRAIL gene and HN gene were connected with a 2A connecting peptide by SOE, and 2691 bp of the TRAIL-2A-HN fusion gene was amplified, which conformed to the expected result (Figure 1).

### Enzyme digestion of recombinant adenovirus plasmids

**Figure 2:**
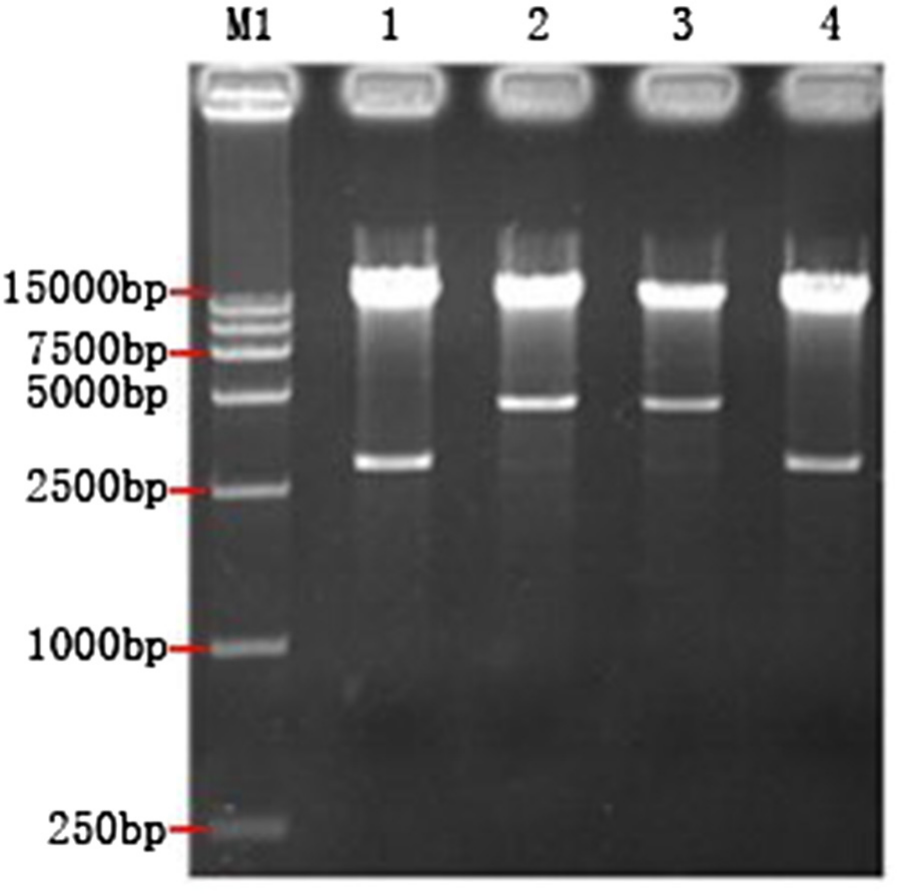
**Identification of recombinant adenoviral plasmids by enzyme digestion.** M: DL15000 DNA Marker; 1: pAd-TRAIL digested by *Pac*I; 2: pAd-HN digested by *Pac*I; 3: pAd-TRAIL-2A-HN digested by *Pac*I; 4: pAd-GFP digested by *Pac*I.

After PacI enzyme digestion of pAd-TRAIL, pAd-HN, pAd-TRAIL-2A-HN, and pAd-GFP, fragments of 3 kb, 4.5 kb, 4.5 kb, and 3 kb were obtained, respectively (Figure 2), indicating that the homologous recombination of pAd-TRAIL and pAd-GFP occurred on the left arm, and that of pAd-HN and pAd-TRAIL-2A-HN occurred at the origin of replication.

### Packaging of adenoviruses and TCID_50_ detection

**Figure 3:**
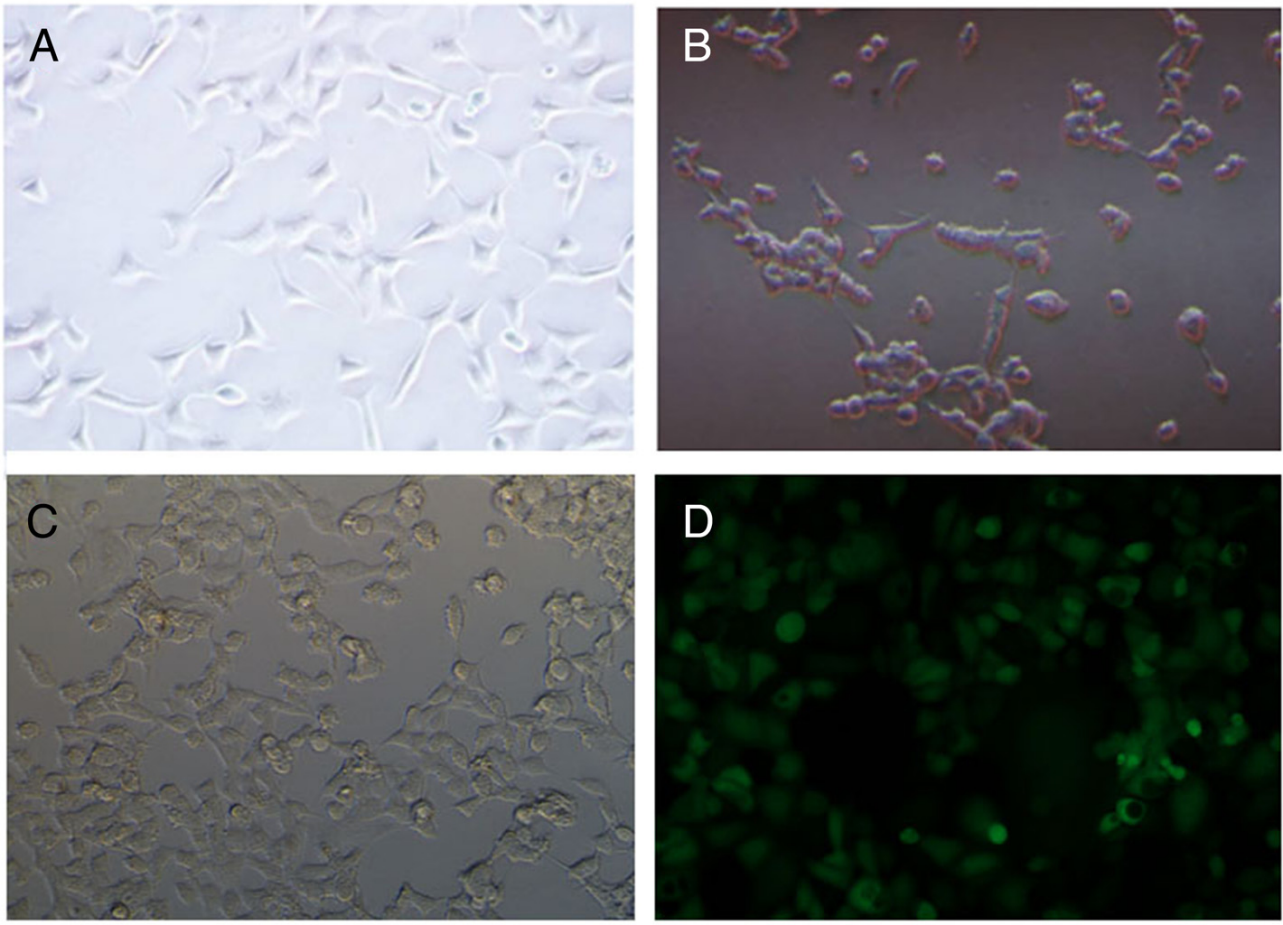
**AD293 cells transfected with recombinant adenoviral plasmids. A:** Normal control AD293 cells; **B:** AD293 cells transfected with recombinant adenoviral plasmids (Ad); **C:** AD293 cells transfected with Ad-GFP (observation in normal light); **D:** AD293 cells transfected with Ad-GFP (observation in fluorescent light). Pictures were taken at 100x magnification.

AD293 cells were cultured by routine methods. When cells in good condition occupied 80%–90% of the culture dish, the recombinant adenovirus plasmids pAd-TRAIL-2A-HN, pAd-TRAIL, pAd-HN, and pAd-GFP were transfected into the AD293 cells. After 7 days, the cells showed pathological changes indicative of a typical cytopathic effect (CPE): the cells became round and karyopyknosis occurred (see Figure 3). The obtained viruses were named Ad-TRAIL-2A-HN, Ad-TRAIL, Ad-HN, and Ad-GFP.

AD293 cells in good condition were inoculated with the viruses, and cellular lesions appeared on the 6th day after inoculation. After purification, the TCID_50_ values of the Ad-TRAIL-2A-HN, Ad-TRAIL, Ad-HN, and Ad-GFP were determined to be 10^9.17^ TCID_50_/mL, 10^9.50^ TCID_50_/mL, 10^9.83^ TCID_50_/mL, and 10^9.50^ TCID_50_/mL, respectively.

### Transfection of MSB-1 cells with recombinant adenovirus and detection of target genes and expressed products

**Figure 4:**
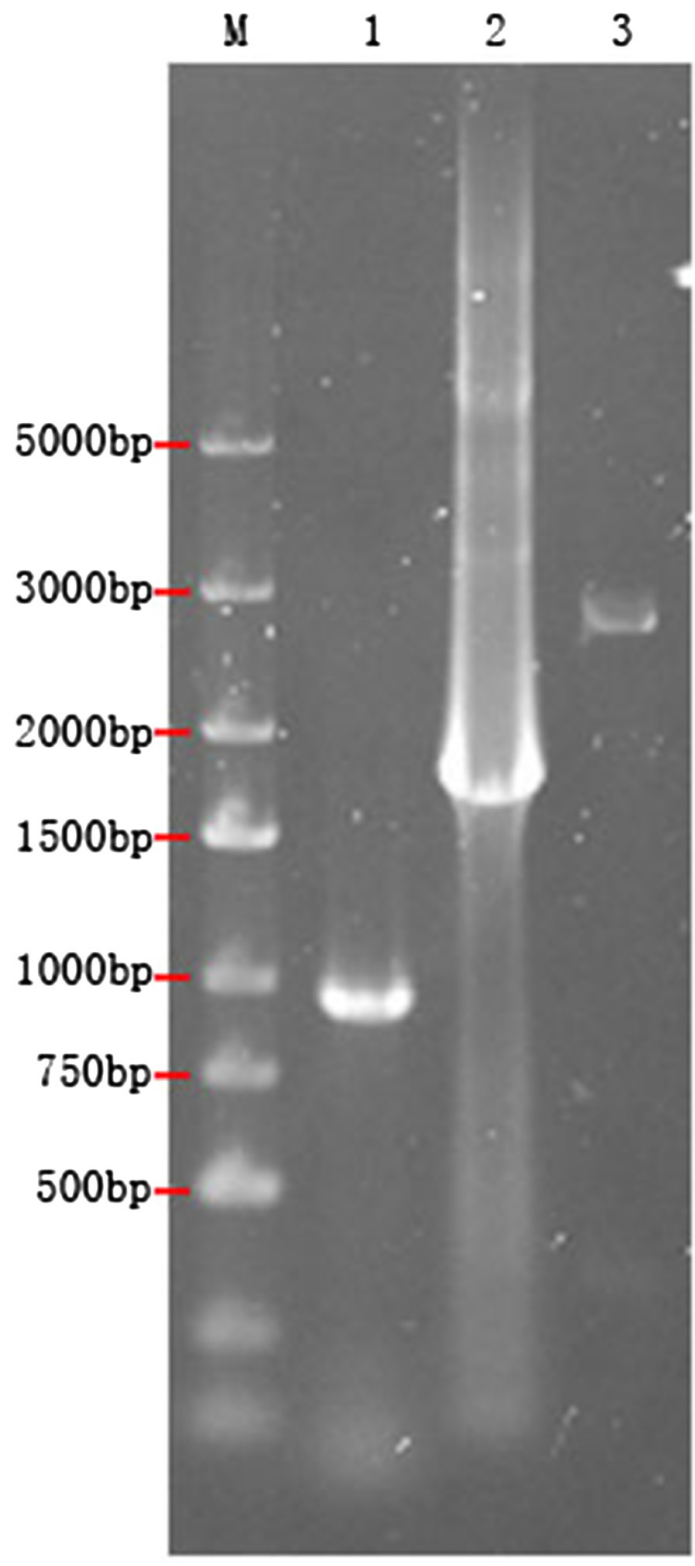
**RT-PCR results for recombinant adenoviral plasmids.** M1: DL5000 DNA Marker; 1: TRAIL gene; 2: HN gene; 3: TRAIL-2A-HN gene.

**Figure 5:**
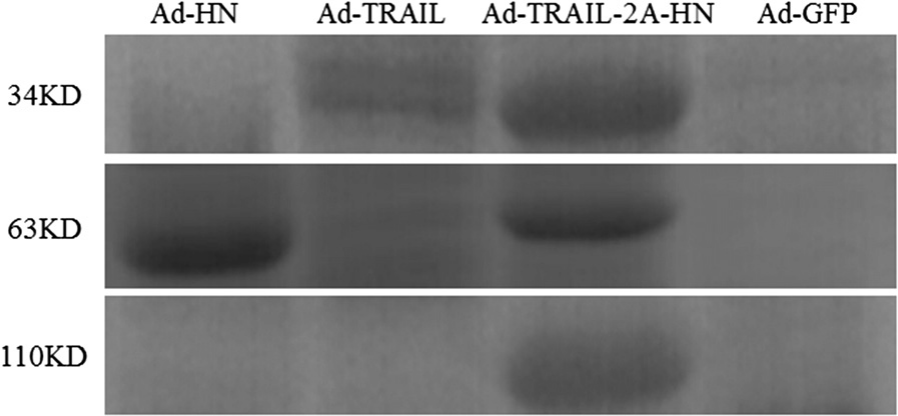
**Protein abundance of TRAIL and TRAIL-2A-HN.**

MSB-1 cells were transfected with Ad-TRAIL, Ad-HN, Ad-GFP, or Ad-TRAIL-2A-HN. After 48 h, the cells were collected and mRNA for target genes was detected with RT-PCR. Results (Figure 4) showed obvious products of 915 bp, 1716 bp, and 2691 bp. The protein expression levels of TRAIL and TRAIL-2A-HN were detected with western blotting (Figure 5). The bands corresponding to TRAIL and TRAIL-2A-HN appeared at 34 kDa and 62 kDa, respectively.

### Influence of recombinant adenoviruses on the growth of MSB-1 tumor cells

**Figure 6:**
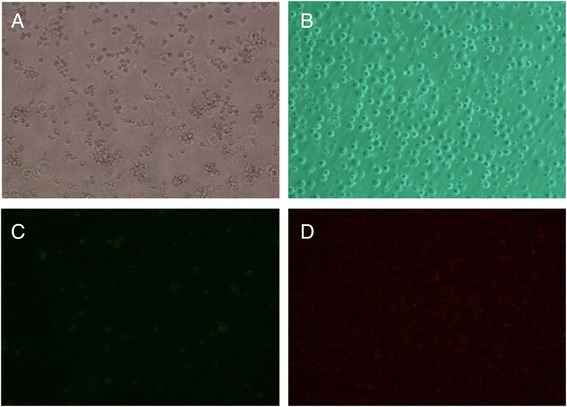
**Apoptosis of MSB-1 cells induced by the recombinant adenovirus. A:** Normal MSB-1 cell; **B:** MSB-1 cell infected by the recombinant adenovirus (observation in normal light); **C:** FITC staining of MSB-1 cell undergoing apoptosis; **D:** PI staining of MSB-1 cell undergoing apoptosis.

MSB-1 cells were infected with recombinant adenoviruses Ad-TRAIL, Ad-HN, or Ad-TRAIL-2A-HN. After 48 h, apoptosis was assayed and changes in morphology were observed using an inverted fluorescence microscope. After infection with Ad-TRAIL, Ad-HN, and Ad-TRAIL-2A-HN, MSB-1 cells showed obvious cellular lesions, such as floating and swelling (Figure 6).

**Figure 7:**
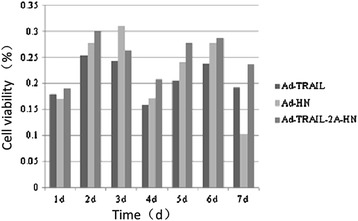
**Inhibitory effect of recombinant adenovirus on MSB-1 cells.** Tumor cells were infected with Ad-TRAIL, Ad-HN, and Ad- TRAIL-2A-HN at 1 MOI. Cell viability was determined using the MTT assay at the indicated time.

The MTT cell viability assay (Figure 7) showed that Ad-TRAIL, Ad-HN, and Ad-TRAIL-2A-HN inhibited the growth of MSB-1 cells. Ad-TRAIL-2A-HN had the strongest inhibitory effect (P < 0.01); Ad-TRAIL had a stable inhibitory effect that was weaker than that of the other viruses (P < 0.01), and Ad-HN showed a moderate inhibitory effect that showed large variation over the duration of the infection (1–7 days) (P > 0.05). Thus, when allowed the same duration of action, the inhibitory effects of the recombinant adenovirus Ad-TRAIL-2A-HN on the growth rate of MSB-1 cells was stronger than that of Ad-TRAIL and Ad-HN.

### Effects of recombinant adenoviruses on MSB-1 cell apoptosis

**Figure 8:**
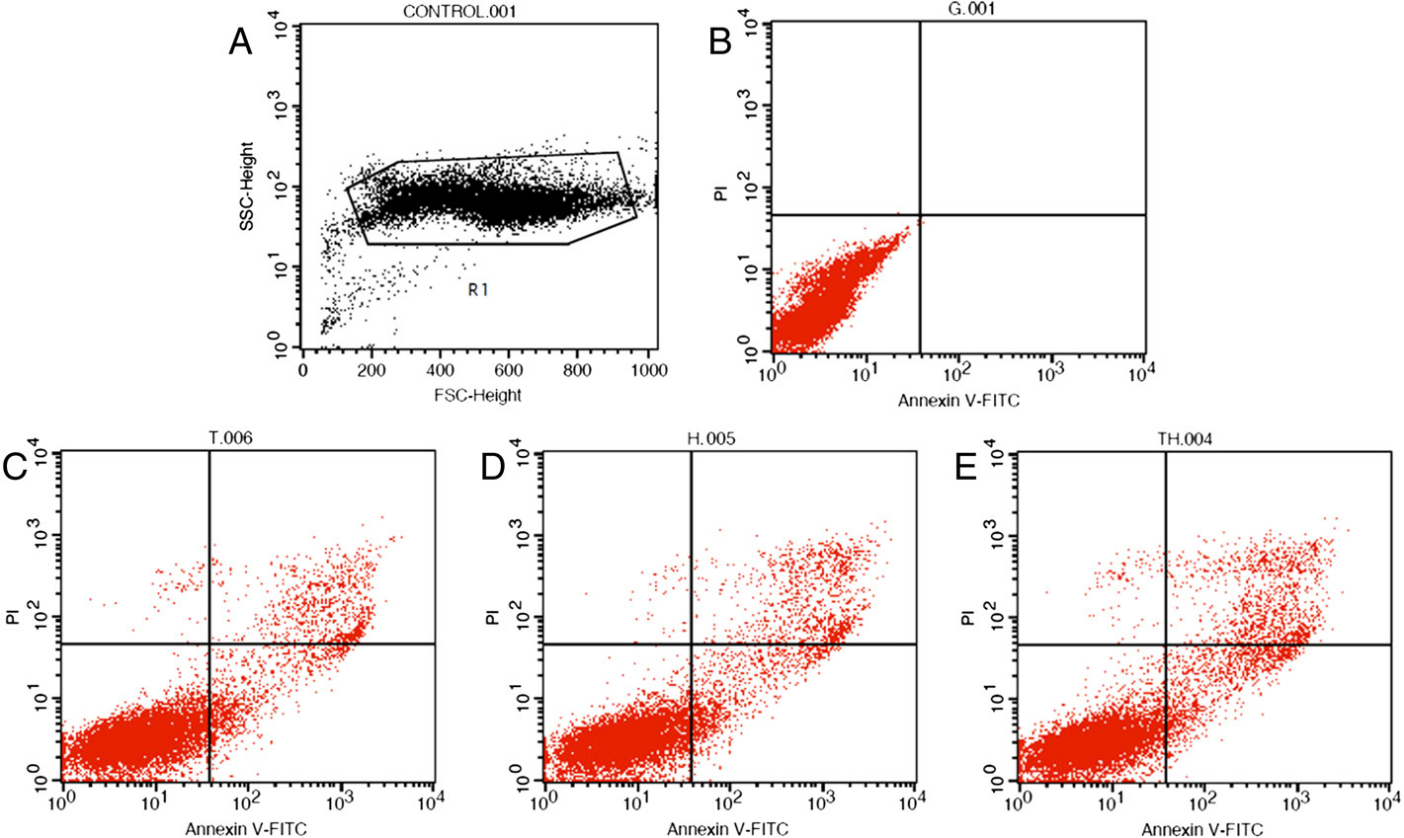
**Flow cytometry of MSB-1 cells infected by the recombinant adenovirus. A:** Test area has been defined before started the assay; **B:** Normal MSB-1 cells without treating adenovirus as a negative control; **C:** MSB-1 cells were treated with Ad-TRAIL, the apoptotic rates produced by Ad-TRAIL was 10.60%; **D:** MSB-1 cells were treated with Ad-HN, the apoptotic rates produced by Ad-TRAIL was 10.15%; **E:** MSB-1 cells were treated with Ad-TRAIL-HN, the apoptotic rates produced by Ad-TRAIL was 11.61%.

After the MSB-1 cells were infected with recombinant adenoviruses, the cells were assayed using the Annexin V FITC/PI kit and flow cytometry, and results are shown in Figure 8. The recombinant adenoviruses accelerated MSB-1 cell apoptosis. The apoptotic rates produced by Ad-TRAIL-2A-HN, Ad-TRAIL, and Ad-HN were 11.61%, 10.60%, and 10.15%, respectively.

## Discussion

Previous reports on the anti-tumor effects of TRAIL or HN have focused on apoptosis induced by either gene alone. In this article, the ability of TRAIL to target tumor cells and the induction of apoptosis by HN were utilized in combination by creating an Ad-TRAIL-2A-HN fusion gene expressing both proteins. Our results indicated that the recombinant adenovirus Ad-TRAIL-2A-HN could be expressed in cell culture, where it inhibited the growth of the MSB-1 Marek’s disease tumor cell line and induced apoptosis. Thus, the results of this study suggest that a recombinant adenovirus expressing a fusion protein of TRAIL and HN produced synergistic inhibition of MSB-1 tumor cells.

TRAIL was first cloned from human peripheral blood lymphocyte cDNA libraries and human heart tissue by Wiley, who also identified its activity [5]. The full length TRAIL gene of the chicken is 1134 bp, and its 912 bp open reading frame has 54.4% homology to that of the human homolog. The extracellular region of TRAIL (894–1134 bp) can effectively inhibit the formation and growth of multiple tumors. In the chicken, TRAIL is highly expressed in the spleen and the mononuclear macrophage system [7]. TRAIL is a member of the tumor necrosis factor (TNF) family, and plays an important role in cell proliferation and the induction of tumor cell apoptosis. TRAIL can bind to certain death receptors to trigger apoptotic signal transduction pathways and induce tumor cell apoptosis, but it has no obvious toxicity or side effects on normal tissues and cells [13-16]. The combined use of TRAIL with chemotherapy, radiotherapy, and proteasome inhibitors can enhance the sensitivity of tumor cells to TRAIL, and thus produce synergistic therapeutic effects against tumor cells. Therefore, the combined use TRAIL with other therapeutic strategies is a promising strategy for anti-tumor therapy [17,18].

The Newcastle disease virus (NDV) HN gene possesses the activities of both hemagglutinin (HA) and neuraminidase (NA). HA enables the virus to adsorb onto the cellular surface and prevents the self-agglomeration of subsequently produced viral particles. NA can hydrolyze sialic acid on the surfaces of host cells to decompose and destroy the receptor. In this way the recognition sites of host cells are exposed, thereby inducing the release of new virus particles from the infected cell membrane. Therefore, in addition to promoting viral adsorption onto tumor cells and invasion of the fusion protein virus, HN hydrolyzes sialic acid, both on the host cell surface and on the sugar chain terminal on the viral particle surface, preventing agglomeration of viral particles on the host cells. Moreover, HN can promote tumor cell adhesion and immunogenicity, which can be recognized by the immune system.

In the immune system, most cells can express TRAIL. After stimulation by type-I interferon IFN-α and type-II interferon IFN-γ, TRAIL expression can be detected in mononuclear macrophages and dendritic cells. Interferon expression is up-regulated in response to viral infection, which induces TRAIL expression [19,20]. In vivo experiments indicated that IFN-γ increased TRAIL expression in natural killer (NK) cells from the rat liver, and thus enhanced the cytotoxicity of NK cells and inhibited the metastasis of tumor cells to the liver [21]. Inactive NDV can also increase the secretion of IFN-α by human peripheral blood monocytes and up-regulate TRAIL expression on the surface of monocytes. However, monoclonal antibodies against HN prevent this effect. Furthermore, by up-regulating the apoptotic ligands of relevant effector cells, HN induces apoptosis in tumor cells [22].

Because TRAIL can selectively induce the apoptosis of tumor cells and has no obvious toxicity to normal cells, recombinant TRAIL represents a potential clinical carcinostatic agent. The structure, inhibitory mechanism, and biological function of TRAIL have been studied with different methods in many laboratories. However, the selective apoptotic effect of TRAIL on tumor cells in comparison to normal cells is not well understood. Therefore, we are both optimistic and cautious with regard to the clinical application of TRAIL.

The administration of NDV to humans will inevitably cause the production of antibodies, and brings the risk of infection and viral diffusion. Moreover, its non-effective components may induce allergic reactions. Therefore, future studies should identify the precise anti-tumor mechanism of HN and develop genetically engineered antineoplastic agents.

In this experiment, the HN gene of the NDV was used to induce apoptosis. HN from NDV was cloned, and the expression product of the recombinant gene was used in place of the whole virus. Thus, the side effects caused by intact viruses, including infection of the organism, were avoided, and the possibility of viral diffusion was precluded. The constructed TRAIL-2A-HN fusion gene combined the apoptosis-inducing function of TRAIL and the adsorptive capacity of HN from NDV for tumor cells, and the capacity of the recombinant adenovirus expressing this fusion gene to induce tumor cell apoptosis was reported. These results provide a basis for future in vivo tumor suppression studies using recombinant adenoviruses.

## Methods

### Amplification of the TRAIL-2A-HN gene

#### Primer design

Two pairs of primers were designed for RT-PCR amplification according to the TRAIL sequence of the chicken (GI:45383401) and the HN cDNA sequence of NDV (GI:124295088) in GeneBank. Based on the sequencing results of the site-directed mutagenesis and fusion genes, the primers were designed as shown in Table 1 and synthesized by Sangon Biotech Co. Ltd. (Shanghai, China).

**Table 1:** **Primers used in the PCR amplification**

**Primer**	**Sequence**
TRAIL-F	5′-GCCGCTACGATGCTGCCCGCG-3′
TRAIL-R	5′-ACCTGCAGGACACAGAATGGCAC-3′
HN-F	5′-ATGGACCGCGCGGTTAACAGAGTC-3′
HN-R	5′-TTAAACCTTATCATCCTTGAGG-3′
CMVtrF	5′-ATCT***GGTACC***ACCATGCTGCCCGCGGGCGGG-3′
CMVtrM	5′-ATCATCTTT*TTGATCAA*CTTTCCCAGGTGCCACTTCACTTGCCAGC
R	AGG-3′
CMVtrMF	5′-ACCTGGGAAAG*TTGATCAA*AAAGATGATGTCAAGAATCCTTCAGG
CMVtrR	AAAAC-3′
2AR	5′-TAGG***CTCGAG***CTAGCTTTGCTTCCTCCAG-3′
	5′-CAT**TGGGCCAGGATTCTCCTCGACGTCACCGCATGTTAGCAGA**
	**CTTCCTCTGCCCTCTCCACTGCC**GCTTTGCTTCCTCCAGAGC-3′
2AF	5′-AGC**GGCAGTGGAGAGGGCAGAGGAAGTCTGCTAACATGCGGT**
CMVhnR	**GACGTCGAGGAGAATCCTGGCCCA**ATGGACCGCGCGGTTAACAG-3′
	5′-TAGG***CTCGAG***TTAAACCTTATCATCCTT G-3′

The bold italics denote *Kpn*I and *Xho*I enzyme cleavage sites; the italics denotes the site-directed mutagenesis of the enzyme cleavage sites *Pac*I; the bold sequence denotes the 2A connecting peptide.

##### Amplification of the TRAIL gene

Specific-pathogen-free (SPF) chickens were chosen for the harvesting of lymphocytes, from which total RNA was extracted with Trizol reagent. The primers TRAIL-F and TRAIL-R (see Table 1) were employed to conduct one-step amplification of the TRAIL gene based on the instructions for the TaKaRa One Step RNA PCR kit (AMV). The sequencing was performed by Sangon Biotech (Shanghai, China). The recombinant plasmid was named pMD-TRAIL after verification.

### Amplification of the HN gene

The lyophilized LaSota strain was dissolved in sterilized physiological saline. After treatment with penicillin and streptomycin, the strains were injected into SPF chicken embryos. Incubation was conducted at 37°C, and the embryos were collected after 4–7 d. The embryos were placed at 4°C for 12 h, and then allantoic fluid was collected from the chicken embryo for extraction of total RNA. As with TRAIL, T-A cloning was employed to amplify the HN gene using the HN-F and HN-R primers (see Table 1). The verified recombinant plasmid was named pMD-HN.

### Construction of the TRAIL-2A-HN fusion gene

#### Site-directed mutagenesis of the TRAIL gene

Analysis of the sequencing results for the TRAIL recombinant plasmid showed that the TRAIL gene contained a PacI enzyme cleavage site, which is indispensable for the construction of an adenovirus vector. Therefore, the TRAIL sequence was modified (TTAATTAA to TTGATCAA) with gene splicing by overlap extension (SOE). The pMD-TRAIL recombinant plasmid was used as a template to amplify the two fragments of the mutated TRAIL gene using two pairs of primers, CMVtrF + CMVtrMR and CMVtrMF + CMVtrR (see Table 1). Reaction system (25 μL): 2.5 μL 10x Pfu Buffer, 2.5 μL MgSO_4_, 0.5 μL d NTPs, 1.0 μL primers (0.5 μL each), 1.5 μL template, 0.5 μL Pfu DNA polymerase, and 16.5 μL ddH_2_O. Reaction conditions: 95°C for 4 min; 30 cycles of 95°C for 30 s, 58°C for 30 s, and 72°C for 1 min; and 72°C for 10 min; followed by gel recovery.

The mutated TRAIL gene was amplified using the purified upstream and downstream TRAIL fragments mentioned above as templates and the CMV trF and CMV trR primers. Reaction system (75 μL): 10 μL MgCl_2_ (15 mmol/L), 2.0 μL dNTPs in 10x PCR Buffer, 4.0 μL each of the upstream and downstream templates, 1 μL long PCR enzyme mix, and 54 μL H_2_O. Reaction conditions: 94°C for 3 min, 95°C for 20 s, 58°C for 30 s, and 68°C for 1 min, for 10 cycles. After adding 2 μL CMVtrF, 2 μL CMVtrR, and 1 μL long PCR enzyme mix, further amplification was conducted under the following conditions: 94°C for 20 s, 58°C for 30 s, and 68°C for 1 min for 25 cycles, followed by a final cycle of 68°C for 10 min. The gel was recovered and purified.

### Amplification of the TRAIL-2A-HN fusion gene

The purified products of the mutated TRAIL fragment were used as a template to amplify TRAIL-2A with the primers CMV trF and 2AR. pMD-HN was used as a template to amplify 2A-HN with the primers 2AF and CMV hnR. The purified TRAIL-2A and 2A-HN were used as templates to amplify TRAIL-2A-HN with the primers CMV trF and CMV hnR. The reaction system and conditions were based on those mentioned above.

### Construction of recombinant adenovirus plasmid pAd-TRAIL-2A-HN

#### Construction of recombinant shuttle vector TRAIL-2A-HN

After the separate double restriction-enzyme digestion by KpnI and XhoI, the recovered TRAIL-2A-HN and pShuttle-CMV were connected with T_4_ DNA ligase at 16°C overnight, and thus the recombinant plasmid pShuttle-CMV-TRAIL-2A-HN was constructed. pShuttle-CMV-TRAIL and pShuttle-CMV-HN were constructed with the same method.

### Construction of recombinant adenovirus plasmid

The plasmids pShuttle-TRAIL-2A-HN and pAdEasy-1 were linearized by the PmeI enzyme digestion and jointly used to transform BJ5183 competent cells. The colonies were screened by PCR of bacteria liquid and identified by PacI enzyme digestion. The positively identified plasmid was named pAd-TRAIL-2A-HN. The recombinant adenovirus plasmids pAd-TRAIL, pAd-HN, and pAd-GFP were constructed using the same method.

### Construction and identification of recombinant adenoviruses Ad-TRAIL-2A-HN, Ad-TRAIL, Ad-HN and Ad-GFP

The recombinant adenovirus plasmid pAd-TRAIL-2A-HN was extracted using the Hispeed Plasmid Purification Kit (Qiagen, Venlo, Netherlands) and digested with PacI enzyme. Lipofectamine 2000 (Invitrogen, Shanghai, China) was employed to transfect the AD293 cells according to the manufacturer’s instructions, and the recombinant adenovirus was obtained after packaging. The mRNA transcription of target genes was detected by RT-PCR, and packaging was performed to acquire the recombinant adenoviruses Ad-TRAIL, Ad-HN, and Ad-GFP. Cell transfection was measured by Ad-GFP using a fluorescence microscope. The viruses amplified at an early stage were purified with CsCl density gradient centrifugation. The titration assay for the virus was carried out with the Ad EasyTM XL Adenovirus Vector System (Stratagene, Shanghai, China) according to the manufacturer’s instructions.

### Cell culture

#### AD293 cell culture

AD293 cells (1 mL) were added to a 25 cm^2^ culture flask (5 × 10^5^ cells/mL), and fresh DMEM culture medium (10% FBS and 1xpenicillin-streptomycin) was added to 5 mL. The culture was conducted at 37°C with a 5% CO_2_ atmosphere and 85% humidity. During passage, the cells were rinsed with 3 mL PBS buffer (room temperature) twice. Digestion was conducted with 0.25% trypsin solution at 37°C for 2–3 min and terminated with 3 mL DMEM culture solution (10% FBS).

### MDCC-MSB-1 cell culture

MSB-1 cells (1 mL) were added to a 25 cm^2^ culture flask (5 × 10^5^ cells/mL) and fresh 1640 culture solution (15% FBS and 1x double antibody) was added to 5 mL. The culture was conducted at 37°C, 5% CO_2_, and 85% humidity. During passage, the cells were rinsed with 3 mL PBS buffer (room temperature) once; after centrifugation at 1000 g for 5 min, the cells were cultured in 1640 culture solution.

### In-vitro expression detection of recombinant adenovirus

#### RT-PCR detection

After MSB-1 cells were transfected by recombinant adenoviruses for 48 h, total RNA of MSB-1 cells was extracted. RT-PCR was conducted to detect the target genes TRAIL-2A-HN, TRAIL, and HN. The reaction condition and system were as previously described for RT-PCR.

### Western blotting

Cultured MSB-1 cells were infected with the recombinant adenoviruses at 100 multiplicity of infection (MOI). The Ad-TRAIL group, Ad-HN group, Ad-TRAIL-2A-HN group, and control group (Ad-GFP) were cultured in an incubator with a 5% CO_2_ atmosphere at 37°C for 48 h, and then the following procedures were performed: protein extraction; SDS-PAGE electrophoresis; protein transfer to a nitrocellulose membrane; blocking of the membrane; dilution of TRAIL and HN antibodies with the sealing solution at a ratio of 1:500; washing the membrane with PBS; reaction with the enzyme-labeled secondary antibody; DAB color development; and photography and recording.

### Evaluation of apoptosis in MSB-1 cells mediated by recombinant adenovirus

#### MTT assay for cell viability

The recombinant adenoviruses were added to the 96-well plate lined with MSB-1 cells at 1 MOI, and cultured at 37°C for 48 h. The cells were treated according to the manufacturer’s instructions., and the optical density at 490 nm was detected with ELISA to determine cell viability.

### MSB-1 cell apoptosis induced by recombinant adenovirus

The recombinant adenoviruses were added to the 96-well plate lined with MSB-1 cells at 1 MOI, and cultured at 37°C for 48 h. Next, the cells were treated according to the instructions of the FITC Annexin V Apoptosis Detection Kit I (Becton Dickinson, Shanghai, China). First, 1–5 × 10^5^ MSB-1 cells, 5 μL Annexin V-FITC, and 5 μL PI (propidium iodide) were added to 1x binding buffer. The reaction was performed in the dark at room temperature for 5–15 min, and flow cytometry was conducted. After the cells were stained with Annexin V-FITC/PI, a drop of the cell suspension was applied to a glass slide. Finally, staining of the cells was observed with a fluorescence microscope.
